# Study of Biochemical Parameters as Predictors for Need of Invasive Ventilation in Severely Ill COVID-19 Patients

**DOI:** 10.2478/jccm-2023-0030

**Published:** 2023-11-14

**Authors:** Azmat Kamal Ansari, Anjali Pitamberwale, Shabana Andleeb Ansari, Tariq Mahmood, Kirti Limgaokar, Geeta Karki, Lalit Singh

**Affiliations:** Shri Ram Murti Smarak Institute of Medical Sciences, Bareilly, Uttar Pradesh, India; Enzene Biosciences, Pune, Maharashtra, India; Teerthanker Mahaveer Medical College and Research Centre, Moradabad, Uttar Pradesh, India; Fergusson College, Pune, Maharashtra, India

**Keywords:** severely Ill COVID-19 patients, prognostic biochemical parameters, invasive ventilatory support, acute respiratory distress syndrome

## Abstract

**Background:**

Though laboratory tests have been shown to predict mortality in COVID-19, there is still a dearth of information regarding the role of biochemical parameters in predicting the type of ventilatory support that these patients may require.

**Methods:**

The purpose of our retrospective observational study was to investigate the relationship between biochemical parameters and the type of ventilatory support needed for the intensive care of severely ill COVID-19 patients. We comprehensively recorded history, physical examination, vital signs from point-of-care testing (POCT) devices, clinical diagnosis, details of the ventilatory support required in intensive care and the results of the biochemical analysis at the time of admission. Appropriate statistical methods were used and P-values < 0.05 were considered significant. Receiver operating characteristics (ROC) analysis was performed and Area Under the Curve (AUC) of 0.6 to 0.7, 0.7 to 0.8, 0.8 to 0.9, and >0.9, respectively, were regarded as acceptable, fair, good, and exceptional for discrimination.

**Results:**

Statistically significant differences (p<0.05) in Urea (p = 0.0351), Sodium (p = 0.0142), Indirect Bilirubin (p = 0.0251), Albumin (p = 0.0272), Aspartate Transaminase (AST) (p = 0.0060) and Procalcitonin (PCT) (p = 0.0420) were observed between the patients who were maintained on non-invasive ventilations as compared to those who required invasive ventilation. In patients who required invasive ventilation, the levels of Urea, Sodium, Indirect bilirubin, AST and PCT were higher while Albumin was lower. On ROC analysis, higher levels of Albumin was found to be acceptable indicator of maintenance on non-invasive ventilation while higher levels of Sodium and PCT were found to be fair predictor of requirement of invasive ventilation.

**Conclusion:**

Our study emphasizes the role of biochemical parameters in predicting the type of ventilatory support that is needed in order to properly manage severely ill COVID-19 patients.

## Introduction

The responsibility of compiling the data on the epidemiology, laboratory diagnosis and management of COVID-19 was handled by the International Federation of Clinical Chemistry (IFCC) and Laboratory Medicine Task Force (LMTF) [1,2]. It was a very important step in the creation of concrete recommendations for diagnosis and treatment of this deadly disease [3,4]. However, these suggestions are frequently based on data from setups with abundant resources while data from resource-limited nations like India is often missing [5].

Acute respiratory distress syndrome (ARDS) is a serious respiratory complication of COVID-19 that necessitates mechanical ventilation [1,2,3,4]. Depending on the severity of the condition, different ventilatory support options may be used for management of COVID-19 [3,4]. Thus, non-invasive and invasive mechanical ventilatory supports are used depending on the situation [3,4]. When predicting respiratory failure and the need for ventilatory support, it is important to take into account a number of variables, such as clinical profile, disease progression, and pre-existing diseases [1,2,3,4,6]. As a result, the majority of models for this purpose are too complicated to be useful [7].

Hospital resources in underdeveloped nations like ours can be severely strained in epidemics like Covid-19 [5,8]. A tool that could accurately anticipate the likely requirement for mechanical ventilation at initial point of contact with the healthcare system, would contribute in better planning and resource allocation [6,7,8,9]. Biochemical parameters correlate well with factors like oxygenation status and the pathophysiological processes linked to unfavourable outcomes. These can not only be used to predict respiratory failure but also the type of ventilatory support that these patients may require in intensive management [6,7,8,9,10,11].

The key components of managing patients with COVID-19 are prompt diagnosis and appropriate intervention [1,2,3,4,11]. It is known that pathophysiological changes related to severe illness are bound to be reflected in terms of the alteration of biochemical parameters. Thus, routine laboratory parameters are used for effective patient monitoring in intensive care [10,13–14]. Based on these we assume that biochemical parameters can predict respiratory failure and thus can provide crucial details about the requirement of type of ventilation in the effective management of COVID-19 patients [6–7,9,10,11,12,13,14]. The majority of the studies on biochemical parameters in COVID-19 patients focus solely on their role as prognostic indicator of mortality. [7,10,11,13] Therefore, we designed this study to examine the utility of biochemical parameters as predictive indicators of requirement of particular type of ventilation in severely ill COVID-19 patients.

## Materials and methods

After receiving approval from Institutional Ethics Committee (IEC) of Shri Ram Murti Smarak (SRMS) Institute of Medical Sciences (IMS), Bareilly, an observational, retrospective cohort study was carried out at our Level 3 COVID Hospital in the Rohilkhand region of Uttar Pradesh, India. We used the information from patients who were admitted to our COVID Intensive Care Unit (ICU) between October 1, 2020, and January 31, 2021. Only severely ill COVID-19 patients who were admitted to our COVID ICU were included in our study. For defining severely ill COVID-19 patients, we utilised the “Revised Guidelines on Clinical Management of COVID-19” published by the Government of India by Ministry of Health & Family Welfare Directorate General of Health Services (EMR Division) [10]. These guidelines define severely ill COVID-19 patients as Laboratory-confirmed (by Real-Time Polymerase Chain Reaction (RT-PCR), TrueNat or Rapid Antigen Test (RAT) COVID-19 cases with at least one of the following:

– Severe pneumonia: Defined as suspicion of having respiratory tract infection, with one or more of the following:
  – Respiratory rate greater than 30 breaths per minute
  – Severe respiratory distress
  – Peripheral capillary oxygen saturation (SPO_2_) less than 90 percent (%) on room air
– ARDS: Defined on the basis of characteristic onset, chest imaging, origin of oedema and oxygenation specified as follows:
  – Onset: Development of new or worsening respiratory symptoms within one week of known exposure
  – Chest imaging: Bilateral opacities not explained by chest imaging studies
  – Origin of oedema: Respiratory failure caused by factors other than cardiac failure or fluid overload
  – Oxygenation: Mild, moderate or severe ARDS: diagnosed on the basis of presence of any of the following:
  – Partial pressure of oxygen in arterial blood (PaO_2_) less than 200 (millimetres of mercury) mmHg or Fraction of inspired oxygen (FiO_2_) less than or equal to 300 mmHg (with positive end-respiratory pressure (PEEP) or Continuous positive airway pressure (CPAP) more than or equal to 5 centimetres (cm) of water (H_2_O)
  – When PaO_2_ is not available, SpO_2_ or FiO_2_ less than or equal to 315
  – Respiratory distress in non-ventilated patient
– Sepsis: Defined as a dysregulated host response to infection resulting in life-threatening organ dysfunction, diagnosed by presence of any of the following:
  – Symptoms like altered mental status, difficult or fast breathing, or skin mottling
  – Signs like low oxygen saturation, reduced urine output, fast heart rate, weak pulse, cold extremities or low blood pressure
  – Laboratory evidence like coagulopathy, thrombocytopenia, acidosis, high lactate, or hyperbilirubinemia
  – Septic shock: Defined as persisting hypotension despite appropriate volume resuscitation, requiring vasopressors in order to maintain mean arterial pressure (MAP) more than or equal to 65 mmHg and serum lactate level less than 2 mmol/L.

The patients suspected to be suffering from COVID-19 without laboratory confirmation, laboratory confirmed COVID-19 cases that do not meet our criteria for severely ill COVID-19, those who were not managed as per Standard Operating Procedures (SOPs) of our ICU due to lack of resources in epidemic or any other reasons (e.g. leave against medical advice) or without sufficient data (e.g. clinical profile, laboratory test results or details of type of ventilation) for further analysis were excluded.

The SOPs of our institute were followed for all patient management procedures, including admission, history taking, physical examination, analysis (conducted either by Point of Care Testing (POCT) devices in the ICU or auto analyzers in the laboratory), and ventilatory management. Data was accessed from patient case files as well as our hospital and laboratory information systems (HIS and LIS).

The patient’s clinical profile (full history, physical examination findings, vital signs from POCT devices, radiological findings, details of the ventilation support provided and diagnosis) as well as the laboratory results from the first arterial blood sample taken after admission at COVID ICU, were all recorded on a specially designed proforma. Our central clinical laboratory’s biochemistry section carried out biochemical investigations (Bilirubin (total, direct, and indirect), Aspartate transaminase (AST), Alanine transaminase (ALT), Alkaline phosphatise (ALP), Lactate dehydrogenase (LDH), Urea, Creatinine, Sodium, and Potassium) while the pathology section estimated D-dimer and Procalcitonin (PCT). Serum interleukin-6 (IL-6) and Ferritin levels were measured in the Chemiluminescence Immunoassay (CLIA) section of our Central Research Laboratory. All parameters were analyzed following the SOPs of the respective sections of our laboratories. Table 1 summarizes the details of the analyzers and the methods (along with their respective system packs) used for all laboratory parameters of present study. Results of quantitative analysis were validated by means of internal and external quality control procedures of our laboratories.

**Table 1.** Instruments and Methods Used for the Quantitative Analysis of Biochemical Parameters:

**Sr. No.**	**Instrument**	**Tests**	**Methods and Reagents**
A.	Mindray BS480 Analyzer (Serum in plain vacutainer)	Total Bilirubin	Diazotized Sulfanilic Acid (DSA) method
		Direct Bilirubin	Diazotized Sulfanilic Acid (DSA) method
		Indirect Bilirubin	Calculated
		Serum Protein	Biuret method
		Serum Albumin	Bromocresol Green (BCG)
		Serum Globulin	Calculated
		Alanine Aminotransferase (ALT)	UV Kinetic assay without PLP (IFCC)
		Aspartate Aminotransferase (AST)	UV Kinetic assay without PLP (IFCC)
		Alkaline Phosphatase (ALP)	para-Nitrophenyl Phosphate and AMP Buffer (IFCC/Kinetic)
		Lactate Dehydrogenase (LDH)	UV Kinetic assay (IFCC)
		Urea	Urease-Glutamate dehydrogenase (GLDH) method
		Creatinine	Sarcosine Oxidase method

B.	Avantor Easylyte Electrolyte Analyzer (Serum in plain vacutainer)	Sodium	Ion-selective electrodes (ISE)
		Potassium	Ion-selective electrodes (ISE)

C.	Beckman Coulter Access 2 Immunoassay System (Serum in plain vacutainer)	IL-6	Chemiluminescent Immunoassay method
		Ferritin	Chemiluminescent Immunoassay method

D.	AQT 90 Flex Immunoassay Analyzer (Whole blood in EDTA Vacutainer)	D-Dimer	Immunoassay
		PCT	Immunoassay

Based on the type of ventilation (invasive or noninvasive) needed for their treatment throughout their stay at our ICU, the study population was split into two groups: patients who underwent invasive mechanical ventilation and patient who were managed by non-invasive mechanical ventilation (NIMV). Patients who received both invasive and non-invasive ventilatory support were considered in the first group (invasive ventilation). To investigate the role of these biochemical parameters in the prediction of the type of ventilation needed in severely ill COVID-19 patients, the results of blood samples taken at the time of admission to the COVID ICU were compared between these groups.

Categorical variables were described as frequency and percentages while continuous variables as mean and standard deviation (SD). Quantitative data were assessed for linearity using Kolmogorov-Smirnov analysis and depending upon the data type appropriate tests of statistical significance (student’s unpaired t-test or Mann-Whitney-U test) were used. Using MedCalc software, the means for continuous variables were compared using independent group p-values. The parameters with p-values < 0.05 were considered statistically significant. Receiver operating characteristics (ROC) curve analysis was used (for all the parameters that showed a statistically significant difference between the two groups) to assess the efficacy of biochemical parameters to predict the requirement of invasive or noninvasive ventilation. AUC of 0.6 to 0.7, 0.7 to 0.8, 0.8 to 0.9, and >0.9 were considered acceptable, fair, good, and excellent discrimination, respectively. Further, for all parameters with an Area Under the Curve (AUC) > 0.6, the sensitivity and specificity as predictors for requirement of invasive or non-invasive ventilation (at specific cut-offs) were also assessed.

## Results

A total of 231 critically ill patients admitted to our COVID ICU of Shri Ram Murti Smarak (SRMS) Hospital between October and January 2021. Based on our criteria we included 178 severely ill COVID-19 patients in the present study.

Out of 178 critically ill patients, 168 (94.38%) patients were diagnosed with Bilateral Pneumonia. All the patients included in our study were given ventilation at the time of admission to the ICU due to severity of disease. Of these, 21 (11.8 %) required invasive ventilation (PRVC mode) while 157 (87.2%) were managed by Non-invasive ventilation. The mortality of patients that were managed on non-invasive ventilation and those who required invasive ventilation was 46% and 95% respectively (Table 2).

The results of biochemical parameters at the time of admission to the COVID ICU in the prediction of the type of ventilation needed in severely ill COVID-19 patients were compared between these groups. The calculated mean, standard deviation and p values of all the parameters under the study are listed in Table 3. The parameters that showed statistically significant difference (p<0.05) between the patients that were managed by non-invasive ventilation as compared to patients those who required invasive ventilation were Urea (p = 0.0351), Sodium (p = 0.0142), Indirect Bilirubin (p = 0.0251), Albumin (p = 0.0272), AST (p = 0.0060) and PCT (p = 0.0420) (Table 3). In patients who required invasive ventilation, the levels of Urea, Sodium, Indirect bilirubin, AST and PCT were significantly higher while Albumin was significantly lower. Other biochemical markers (Creatinine, Potassium, Total and Direct bilirubin, Total Protein, Globulin, ALT, ALP, LDH, , IL-6, D-Dimer and Ferritin did not differ significantly between the two groups (p-value > 0.05).

On ROC analysis (based on our criteria AUCs of 0.6 to 0.7, 0.7 to 0.8, 0.8 to 0.9, and >0.9, respectively as acceptable, fair, good, and exceptional for discrimination as prediction) we found that Albumin, Sodium, PCT and D-Dimer were found to be promising prognostic markers. Higher levels of Albumin was found to be acceptable indicator of maintenance on non-invasive ventilation while higher levels of Sodium and PCT were found to be fair predictor of requirement of invasive ventilation in intensive care of severely ill COVID-19 patients.

**Fig.1.**
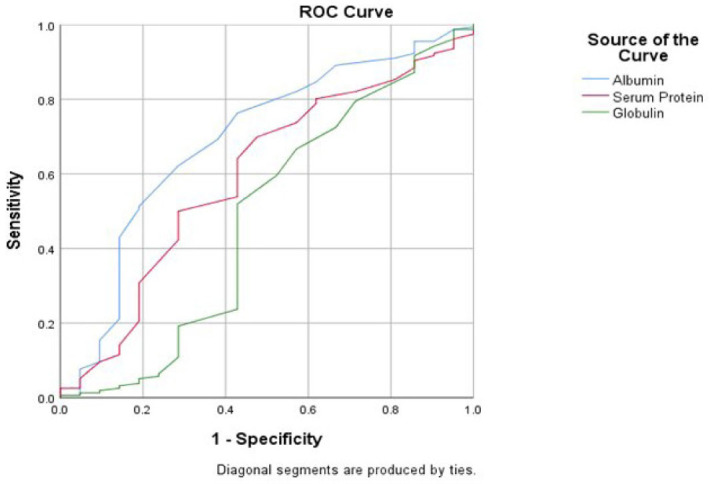
ROC Curve of Total Protein, Albumin and Globulin as a predictor of Non-invasive Ventilation

The ROC curve for Albumin as an indicator of maintenance on non-invasive type of Ventilation has an AUC of 0.692 (p = 0.0272) which puts it on the upper bounds of an acceptable score. For a cut off value of 3.05 g/dl of Albumin, the sensitivity and specificity are 0.763 and 0.571 respectively (Figure 1).

**Table 2.** Main Characteristics Severely Ill COVID-19 Patients.

**X-ray findings**	**Bilateral Pneumonia (n=168)**	**Without Bilateral Pneumonia (n=10)**

Males	126 (71%)	7 (4%)
Females	42 (24%)	3 (1%)

**Type of Ventilation**	**Survived (n= 86)**	**Expired (n=92)**

Invasive	1 (1% out of total population)	20 (11% out of total population)
Non-Invasive	85 (48% out of total population)	72 (40% out of total population)

**Table 3.** Comparison of Biochemical Parameters Between the Groups of Severely Ill COVID-19 Patients According to the Type of Ventilatory Support They Needed for the Intensive Care

**Parameters**	**Non-invasive Ventilation**		**Invasive Ventilation**		**p-value**

	**Mean**	**Standard Deviation**	**Mean**	**Standard Deviation**	
Urea (mg/dl)	71.98	51.71	98.09	61.53	0.0351
Creatinine (mg/dl)	1.61	1.66	1.76	0.93	0.6845
Na^+^(mmol/l)	133.84	14.7	142.00	9.31	0.0142
K^+^ (mmol/l)	4.49	2.84	4.36	1.00	0.8356
T-Bil (mg/dl)	0.88	1.78	1.25	1.45	0.3620
D-Bil (mg/dl)	0.54	1.43	0.65	0.85	0.7310
Ind-Bil (mg/dl)	0.33	0.46	0.60	0.87	0.0251
Serum Protein (g/dl)	6.34	0.94	6.17	0.81	0.4272
Albumin (g/dl)	3.36	0.57	3.06	0.66	0.0272
Globulin (g/dl)	3.22	2.23	4.29	5.27	0.0952
AST (IU/L)	70.55	61.59	116.95	125.95	0.0060
ALT (IU/L)	56.02	46.93	67.99	136.89	0.4211
ALP (IU/L)	124.22	117.72	154.00	93.68	0.2676
LDH (IU/L)	526.42	265.67	543.67	244.03	0.7783
PCT (ng/ml)	2.47	7.95	6.45	11.11	0.0420
IL-6 (pg/ml)	550.75	2818.64	246.29	318.84	0.6224
D-Dimer (µg/ml)	6407.22	14105.46	9058.29	7715.01	0.4003
Ferritin (ng/ml)	805.80	931.27	1053.73	968.32	0.2556

**Fig. 2.**
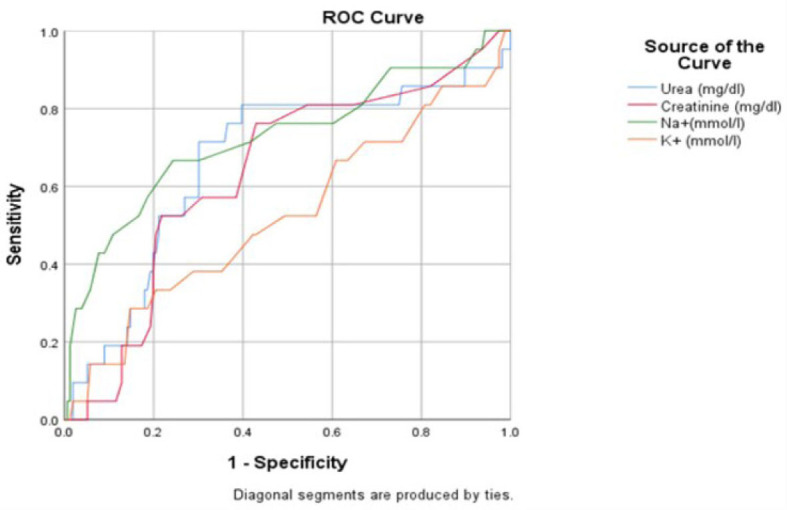
ROC curve for Urea, Creatinine, Sodium and Potassium as an indicator of Invasive Ventilation

The ROC curve for Sodium as a predictor of requirement of Invasive type of Ventilation during the intensive care of severely ill COVID-19 patients has an AUC of 0.724 (p = 0.0142) which puts it on the lower bounds of a fair score. For a cut off value of 138 mmol/l of Sodium, the sensitivity and specificity are 0.667 and 0.756 respectively (Figure 2).

**Fig. 3.**
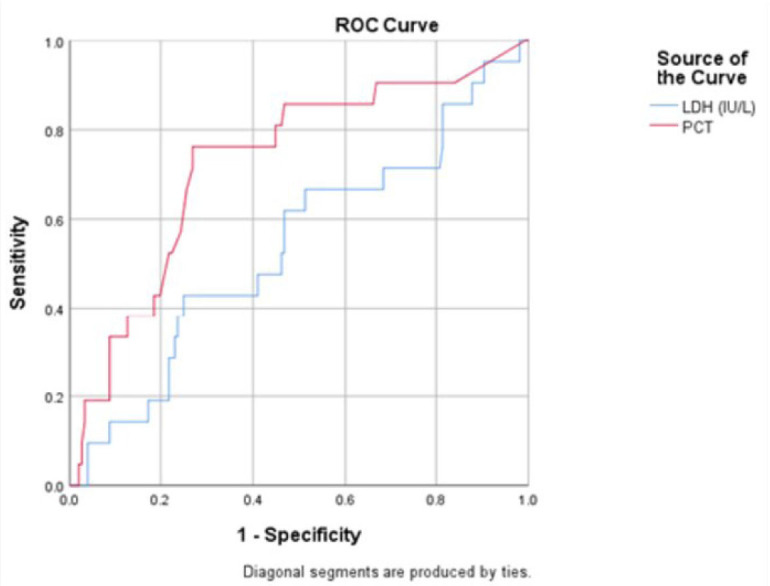
ROC curve for LDH and PCT as an indicator of Invasive Ventilation

On ROC analysis for PCT as an indicator of requirement of Invasive type of Ventilation, it has an AUC of 0.725 (p = 0.0420) which puts it on the lower bounds of a fair score. For a cut off value of 0.76 ng/ml of PCT, the sensitivity and specificity are 0.762 and 0.707 respectively (Figure 3).

**Fig. 4.**
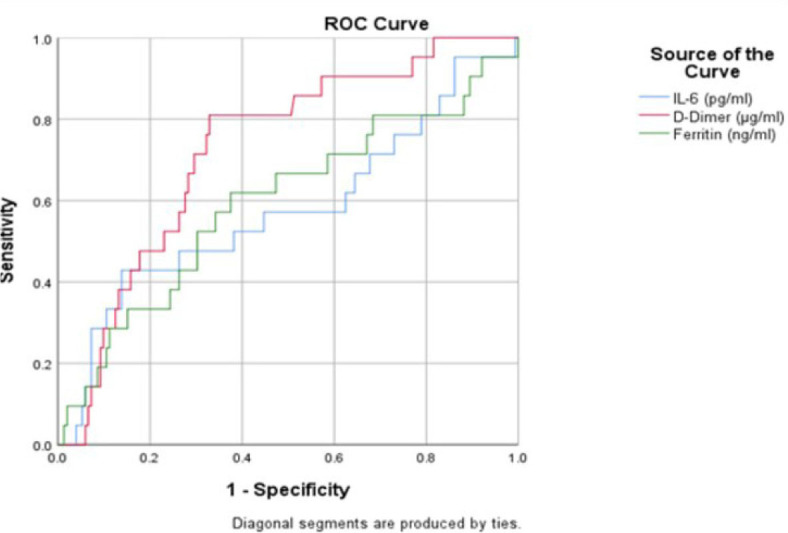
ROC curve for IL-6, D-dimer and Ferritin as an indicator of Invasive Ventilation

The ROC curve for D-dimer as an indicator of requirement of Invasive type of Ventilation has an AUC of 0.727 (p = 0.4003) which puts it on the lower bounds of a fair score. For a cut off value of 3230 µg/ml of D-dimer, the sensitivity and specificity are 0.810 and 0.660* respectively (Figure 4).

## Discussion

At the time of ICU admission, the severely ill COVID-19 patients in our study were found to have distinctive clinical and laboratory profiles. The majority (94.38%) of the patients in our study had bilateral pneumonia. The patients with severe respiratory issues are frequently intubated [11,14]. In our study, only 11.8% of the patients required intubation but those who did had very significant death rates. Therefore, invasive ventilation and mortality were found to be positively correlated in our study [11,14–15]. Our research demonstrates a connection between invasive ventilation and unfavourable results (95% mortality in patients with invasive ventilation while 46% mortality in patients managed non-invasively). Previous research has demonstrated that the need for invasive ventilation may be brought on by hypoxia [6,9,11,14]. Our study conciliates with this data.

The parameters that showed statistically significant difference (p<0.05) between the patients that were managed by non-invasive ventilation as compared to patients those who required invasive ventilation are Urea (p = 0.0351), Sodium (p = 0.0142), Indirect bilirubin (p = 0.0251), Albumin (p = 0.0272), AST (p = 0.0060) and PCT (p = 0.0420). In patients who required invasive ventilation, the levels of Urea, Sodium, Indirect bilirubin, AST and PCT were higher while Albumin was lower. Several studies have reported the association of these parameters with mortality. Since these parameters are related to adverse outcomes, their role in predicting requirement of type of ventilation is conciliating with earlier studies [6–7,9,10,11,16,17,18,19,20,21,22,23,24,25,26,27,28,29].

Other biochemical markers (Creatinine, Potassium, Total and Direct bilirubin, Total Protein, Globulin, ALT, ALP, LDH, , IL-6, D-Dimer and Ferritin did not differ significantly between the two groups (p-value > 0.05). Few studies have proposed these parameters as predictor of mortality but in the present study these parameters failed to correlate with the requirement of type of ventilation [10–11,19,20,21,22,26,27,28,29].

On ROC analysis, we found that Sodium, PCT and D-Dimer were found to be promising prognostic markers. To our surprise despite of statistical differences between the levels of Urea, Indirect bilirubin and AST among the two groups, they were not proven to be a promising prognostic marker on ROC analysis. However, D-Dimer was found to be promising prognostic marker on ROC analysis despite of the fact that its levels were not significantly different among the two groups.

Urea levels were found to be statistically different between the groups of patients that were managed by non-invasive ventilation as compared to those who required invasive ventilation (p = 0.0351). However, on ROC analysis it failed to establish its role as predictor of type of ventilation in severely ill COVID-19 patients. Many studies focusing on correlation of renal function tests on adverse outcomes in COVID-19 have reported such conflicting observations [11,19,20,21].

Our results indicated that sodium levels (p = 0.0142) are a predictor of the need for invasive ventilation with a ROC cut off value of >138 mmol/L (AUC 0.724). Previous research has linked dysnatremia to poor outcomes and longer stays in the intensive care unit [19,20,21]. The specific cause of the elevated sodium levels and their contribution to the pathophysiology of the illness are yet unknown [21]. However, a number of studies suggest that COVID-19 patients’ electrolyte state needs to be given additional attention [20–21].

Indirect bilirubin as well as AST levels were found to be statistically different between the groups of patients that were managed by non-invasive ventilation as compared to those who required invasive ventilation (p = 0.0251 and p = 0.006, respectively). However, on ROC analysis they failed to establish their role as predictor of type of ventilation in severely ill COVID-19 patients. Very few studies focusing on correlation of liver function tests on adverse outcomes in COVID-19 have reported such conflicting observations [10,13,26,27,28,29].

In our study the demand for invasive ventilation in intensive management of severely ill COVID-19 patients was fairly predicted by the ROC Curve of PCT >0.76 ng/ml (AUC 0.725). Several studies have suggested that PCT may be a useful in identifying COVID-19 individuals who are at a high risk of clinical deterioration [17,18].

The difference in D-dimer’s values was not found to be statistically significant (p=0.4003) between the two groups in our investigation. However, ROC analysis of D-dimer suggests that it is a fair predictor of the need for invasive ventilation along the course of the disease as well as a predictor of mortality with a cut off value of >3230 g/ml (AUC 0.721). Due to its high sensitivity (0.810) but relatively low specificity (0.660), we suggest that measuring D-dimer levels at the time of admission can be a useful screening tool to identify patients who will need intubation throughout their ICU stay. Review of literature reveals that D-dimer is a very useful predictive tool of adverse outcome in Covid-19 [22,23,24,25].

Based on the results of our study, we suggest that biochemical parameters be used as a practical predictor of the need for a specific kind of ventilation in critically ill COVID-19 patients due to the routine monitoring of these indicators. We believe that common biochemical parameters can be utilised as reliable indicators to forecast the kind of ventilation needed in COVID-19 patients who are critically ill [10,16,17,18,19,20,21,22,23,24,25]. Patients with COVID-19 who are more likely to experience respiratory failure can be handled effectively with the aid of readily available prognostic markers [4,9,11]. The efficient use of the existing resources can be increased with prompt prognostication and interventions [10]. Utilising resources wisely is crucial, particularly during pandemics like COVID-19 especially in developing countries like ours [8,10].

The conception and execution of this study at an ICU renowned for its high-quality care and adherence to its protocols is its main strength. Some of the advantages of our study include its scientific design (appropriate inclusion and exclusion criteria, adequate sample size, and statistical analysis by appropriate methods), the inclusion of the findings of the investigation conducted at a particular time (when clinical syndromes associated with severe COVID-19 were identified), and the quantitative analysis of all biochemical parameters using state-of-the-art techniques. Moreover, as the study concentrated on the use of feasible biochemical parameters that are easily accessible at the majority of critical care units, it is really very useful for resource limited setups. During the pandemic we have realised that it is impossible to use complex predictive models in the high patient load environment. Hence in such scenario it makes sense to earmark certain routine parameters which have a high predictive value. More research into the prognostic significance of the other patient related factors and laboratory parameters may yield more hints.

This study’s primary drawback is its retrospective design. However, we assume that the accuracy of the results is acceptable as the study was well planned and executed,. Some other drawbacks are small sample size and the inclusion of patients with pre-existing illnesses. The biggest challenge with prediction in diseases like COVID-19 is that the condition of these patients is highly dynamic and day to day factors can affect the patient outcome. Although the idea of using biochemical parameters recorded at the admission for identification of patients at higher risk of deterioration and thus the need for more aggressive treatment modalities is theoretically very promising. It is extremely difficult to build a flawless prediction framework using only such simple parameters. Surely the inclusion of additional variables in the prediction requirement invasive ventilation would be more scientific but the aim of present study was to assess the utility of biochemical parameters as feasible predictors for the need of invasive ventilation in severely ill COVID-19 patients.

## Conclusion

Biochemical parameters, especially Urea, Sodium, Indirect bilirubin, AST, PCT and Albumin can be used to predict the requirement of type of ventilation in management of severely ill COVID-19 patients. We propose that the monitoring of these biochemical parameters can facilitate in the prognostication of respiratory failure and the requirement of type of ventilation in management of severely ill COVID-19. Prognostication is very important in resource limited setups and thus helpful to improve the clinical management of these high-risk patients by adequate planning. However, large-scale multi-center studies to evaluate the utility of biochemical parameters in the prediction of respiratory compromise of COVID-19 and the requirement of type of ventilation in management of severely ill patients, are required to validate our findings. It would really be very useful if these easy available biochemical parameters are validated to be prognostic markers in severely ill COVID-19 patients.
